# The Effect of Camphor and Borneol on Rat Thymocyte Viability and Oxidative Stress

**DOI:** 10.3390/molecules170910258

**Published:** 2012-08-27

**Authors:** Emiliya Cherneva, Voja Pavlovic, Andrija Smelcerovic, Denitsa Yancheva

**Affiliations:** 1 Department of Chemistry, Faculty of Pharmacy, Medical University of Sofia, 2 Dunav Str., 1000 Sofia, Bulgaria; 2 Institute of Physiology, Faculty of Medicine, University of Nis, Bulevar Dr Zorana Djindjica, 18000 Nis, Serbia; 3 Department of Pharmacy, Faculty of Medicine, University of Nis, Bulevar Dr Zorana Djindjica 81, 18000 Nis, Serbia; 4 Institute of Organic Chemistry with Centre of Phytochemistry, Bulgarian Academy of Sciences, Acad. G. Bonchev Str., Build 9, 1113 Sofia, Bulgaria

**Keywords:** camphor, borneol, cytotoxicity, oxidative stress, mitochondrial membrane potential

## Abstract

Camphor and borneol are wildly distributed in the essential oils of medicinal plants from various parts of the World. Our study has been carried out to evaluate the effect of these two bicyclic monoterpenes on rat thymocytes. Camphor and borneol at concentrations of 0.5 and 5 µg/mL did not induce significant toxicity on the immune system cells, while a significant increase of thymocyte viability was detected when cells were incubated with 50 µg/mL of camphor. A significant increase of cell viability was similarly detected when thymocytes were cultivated with borneol at concentrations of 0.5 and 5 µg/mL. The role of camphor and borneol in reactive oxygen species (ROS) production and mitochondrial membrane potential (MMP) disturbances in rat thymocytes as well as their potential mechanism(s) of action were also discussed.

## 1. Introduction

**Scheme 1:**
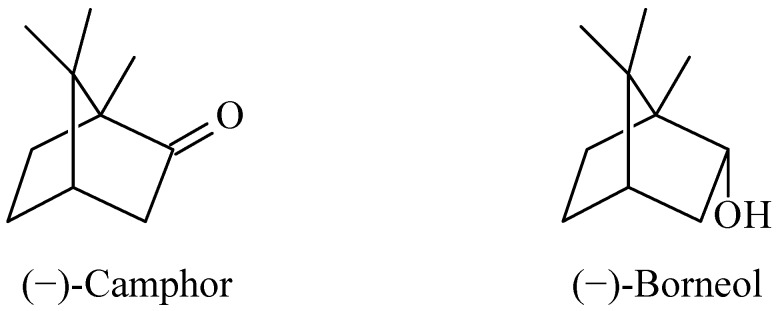
Chemical structures of the compounds under study.

Terpenoid compounds are isolated from a variety of organisms and constitute a large class of natural products. Many terpenoids have biological activities and are used for medical purposes. Camphor (C_10_H_16_O; 4,7,7-trimethylbicyclo[2.2.1]heptan-3-one) and borneol (C_10_H_18_O; 4,7,7-trimethylbicyclo[2.2.1]heptan-3-ol) (Scheme 1) are bicyclic monoterpenes found widely in plants. Croteau suggested the biosynthesis pathways of camphor, where in the last step borneol is oxidized to camphor [1]. Prasad *et al*. have observed that under conditions of low oxygen, *Pseudomonas putida* and isolated P450_cam_ reduce camphor to borneol [2].

(−)-Camphor is one of the oldest known organic compounds and is one of the major constituents of the essential oil from Dalmatian sage [3]. Camphor’s purported medicinal benefits currently include local anesthetic, antipruritic, antiseptic, and mild expectorant activity [4]. It has a characteristic camphoraceous odor and is used commercially as a moth repellent and as a preservative in pharmaceuticals and cosmetics [5]. Borneol, a widely used food and cosmetics additive, has analgesic, anti-inflammatory, antibacterial and penetration enhancing effects [6]. 

For much of the last century, terpenes were depicted as products of detoxification or overflow metabolism [7]. However, starting in the 1970s, a number of terpenes were demonstrated to be toxins, repellents or attractants to other organisms, which led to the belief that they have ecological roles in antagonistic or mutualistic interactions among organisms [8]. Camphor remains a product with the potential for serious toxicity in the pediatric patient. In children less than 6 years of age, exposure to 500 mg or more requires rapid triage to the closest health care facility [4]. The toxicologic assessment of borneol was reviewed recently [9,10]. Since the effect of camphor and borneol on rat thymocytes has not been reported, the current study was designed to evaluate the toxic effect and potential mechanisms involved of these two compounds on cells of the immune system. 

## 2. Results and Discussion

To evaluate the cytotoxicity of camphor and borneol in rat thymocytes, cells were incubated with increased concentrations (0.5, 5, 50 µg/mL) of each compound for a period of 24 h. The current study results show that camphor application of 50 µg/mL resulted significantly increased thymocyte viability (Table 1). On the other hand, as evaluated by CCK-8 assay, rat thymocyte incubation with borneol (0.5 and 5 µg/mL) showed significantly increased cell viablity (Table 1). However, as given in Table 1, significant increase of rat thymocytes viability was not detected when cells were incubated with highest concentration (50 µg/mL) of borneol. 

**Table 1:** The effect of camphor and borneol on rat thymocytes toxicity.

Compound	Concentration (µg/mL)	Cytotoxicity (CCK-8 assay)
	0.5	0.927 ± 0.132
Camphor	5	1.024 ± 0.083
	50	1.111 ± 0.033 *
	0.5	1.067 ± 0.004 **
Borneol	5	1.070 ± 0.015 **
	50	0.975 ± 0.144
Control cells		0.999 ± 0.005

Rat thymocytes (5 × 10^5^ cells/well) were cultivated with increasing concentrations of camphor and borneol (0.5, 5, 50 µg/mL) for 24 h and cell toxicity was evaluated as described in the section Materials and methods. Results are presented as mean percentage ± SD. Absorbances were presented as ratio for comparison with control samples. Abbreviations: ** *p* < 0.01, * *p* < 0.05 compared to control (non-treated) cells.

The involvement of oxidative stress in rat thymocyte toxicity was assessed by testing the effect of camphor and borneol on intracellular ROS production, by using the fluorescent probe H2DCF-DA. As shown in Table 2, rat thymocytes incubation with camphor resulted in significant increase of intracellular ROS production in all tested concentrations. 

**Table 2:** The effect of camphor and borneol on ROS production in rat thymocytes.

Compound	Concentration (µg/mL)	Intracellular ROS production
	0.5	1.413 ± 0.068 ***
Camphor	5	1.399 ± 0.093 **
	50	1.251 ± 0.049 *
	0.5	1.268 ± 0.102
Borneol	5	1.224 ± 0.108
	50	1.164 ± 0.191
Control cells		1 ± 0.042

Cells (5 × 10^5^ cells/well) were treated with increasing concentrations of camphor and borneol (0.5, 5, 50 µg/mL) for 24 h and intracellular ROS production was evaluated by using redox-sensitive probe (H2DCF-DA), as described in the section Materials and methods. Results are presented as ratio of mean fluorescence intensity ± SD for comparison with control samples. Abbreviations: *** *p* < 0.001, ** *p* < 0.01, * *p* < 0.05 compared to control (non-treated) cells.

Cell treatment with increasing borneol concentrations was not able to induce significant increase of intracellular ROS production, even there is slight ROS increase detected in all rat thymocyte cultures (Table 2). As given in Table 2, minimal intracellular ROS production was observed when cells were cultured with 50 µg/mL of borneol. Since the alteration of mitochondrial membrane represents one of the early events in cytotoxicity, we next examined the changes of MMP in rat thymocytes after cultivation with camphor and borneol. Analysis of intracellular Rhodamine 123 fluorescence showed that rat thymocyte exposure to camphor resulted in decreased MMP, but a statistically significant decrease (*p* < 0.01) was detected when cells were cultured with 0.5 µg/mL of camphor (Table 3). Similar results of altered MMP were obtained when cells were incubated with increasing borneol concentrations. Significant MMP decrease (*p* < 0.001) was verified when rat thymocytes were cultured with 0.5 µg/mL of borneol (Table 3), as evaluated by fluorescence intensity of intracellular Rhodamine 123. 

**Table 3:** The effect of camphor and borneol on mitochondrial membrane potential of rat thymocytes.

Compound	Concentration (µg/mL)	Mitochondrial membrane potential
	0.5	0.740 ± 0.071 **
Camphor	5	0.919 ± 0.047
	50	0.925 ± 0.132
	0.5	0.799 ± 0.022 ***
Borneol	5	0.856 ± 0.084
	50	0.876 ± 0.110
Control cells		0.999 ± 0.076

Cells (5 × 10^5^ cells/well) were treated with increasing concentrations of camphor and borneol (0.5, 5, 50 µg/mL) for 24 h and mitochondrial membrane potential was evaluated by using Rhodamine 123, as described in the section Materials and methods. Results are presented as ratio of mean fluorescence intensity ± SD for comparison with control samples. *** *p* < 0.001, ** *p* < 0.01 compared to control (non-treated) cells.

Monoterpenes are the primary components of plant essential oils and the effects of many medicinal herbs have been attributed to them [11,12]. Camphor and borneol are common drugs for skin irritation diseases and have also used for analgesia and as anesthetic agents in traditional Chinese and Japanese medicine [13,14]. Even though it has been shown earlier that camphor and borneol have significant antimicrobial activity [15], represent potent inhibitors of nicotinic acetylcholine receptors that belong to the ionotropic ligand-gated ion channels [16] and inhibit osteoclast activity *in vitro* [17], the possible effects of these compounds on the cells of the immune system are largely unknown. The results obtained in our study demonstrate that camphor was not able to induce significant reduction of cell viability, while the maximal camphor concentration used in our study markedly increased thymocyte viability, after 24 h of incubation. On the other hand, cell exposure to increasing borneol concentrations resulted in significantly increased viability, except when the highest borneol concentrations was used (50 µg/mL). Also, to our knowledge this is the first report indicating the effect of tested compounds on the primary cells of the immune system, indicating their potential usage as a natural health product for camphor at higher and borneol at lower concentrations. Similar results were documented in a recent study [18] showing no significant toxic effect of camphor and borneol on human acute monocytic leukemia cell line (THP-1). However, it should be mentioned that the non-cytotoxic effect of camphor and borneol was documented by testing the essential oil, using lower camphor and borneol concentration than we used in our study and evaluated by a trypan blue viability assay [18]. Cytotoxic effects of borneol in different rat cells [19] and in different cell lines [20] were documented earlier, as well as protective effect of borneol against exogenous oxidative DNA damage [21]. Taking into account the previous findings, in the next set of experiments we tried to determine the potential role of camphor and borneol on ROS production and MMP disturbances in rat thymocytes. Analysis of H2DCF-DA fluorescence intensity revealed that cell incubation with camphor resulted in significantly increased ROS production in all tested cultures, while significantly decreased MMP was detected only in a cell culture with maximal ROS production, indicating that ROS production and altered MMP may not be limiting factors in the induction of cell toxicity by camphor. On the other hand, borneol application in cell cultures was not able to induce significantly increased ROS production. Markedly decreased MMP was observed only in cell cultures with minimal borneol concentration, showing that neither ROS production nor MMP disturbances appear to be involved in thymocyte toxicity induced by borneol. It is well documented that immune cells are sensitive to oxidative stress because of the high content of polyunsaturated fatty acids in their plasma membranes and a high production of ROS, which is part of their normal function [22]. Intensive ROS production may induce apoptosis by oxidative stress or direct ROS damage to cellular components [23,24,25]. Cell death depends, in part, upon mitochondrial dysfunction, which if often characterized by increased production of ROS, increased membrane permeability and eventual release of cell death mediators from mitochondria [26]. It has been postulated for long time that apoptosis and necrosis are functionally and morphologically distinct forms of cell death. However, this concept was changed due to observation that cells triggered to undergo apoptosis will dye by necrosis when the intracellular ATP levels are depleted [27]. Taking into account previous finding we can speculate that camphor and borneol application to cell culture may not induce ROS generation crucial for cell death, but in certain concentrations may lead to intracellular low energy levels with resulting cytotoxicity, but this hypothesis requires further studies which are currently in progress.

## 3. Experimental

### 3.1. Animals

Experiments were performed on adult male Wistar rats (150−180 g), 8−10 weeks old, bred at the Vivarium of the Institute of Biomedical Research, Medical Faculty, Nis, Serbia, under conventional laboratory conditions and in accordance with national animal protection guidelines.

### 3.2. Materials

Culture medium (CM) was prepared using RPMI 1640 (Sigma, St. Louis, MO, USA), according to the manufacturer’s instructions. CM containing 25 mM HEPES, 2 mM glutamine, penicillin (100 U/mL), streptomycin (100 μg/mL) and 10% fetal calf serum (FCS). 2',7'-Dichlorofluorescin diacetate (H2DCF-DA), Cell Counting Kit (CCK-8) and Rhodamine 123 were purchased from Sigma-Aldrich. (−)-Camphor (purity ≥95.0%) and (−)-borneol (purity ≥99.0%) were purchased from Fluka (Buchs, Switzerland).

### 3.3. Preparation of Thymocytes

Rat thymocytes were isolated as described previously [28,29]. The viability of the isolated cells, as determined by trypan blue dye exclusion test, was always over 95%. Isolated thymocytes were counted and adjusted to a density of 5 × 10^6^ cells/mL of CM. 

### 3.4. Cell Culture

Isolated thymocytes were cultivated in 96-well round-bottom plates (NUNC, Aarhus, Denmark), containing a 100 µL of cell suspension (5 × 10^5^ cells) in each well. Cells were treated with increasing concentrations (0.5, 5, 50 µg/mL) of camphor and borneol diluted in appropriate amounts of 96% ethanol. Control samples were cultured in CM, with appropriate amounts of 96% ethanol (9.6%). Concentrations of tested substances used in our study were adopted from our previous report [29], regarding the effect of different compounds on thymocytes toxicity. All cell cultures were done in triplicates and cultivated for 24 h in an incubator (Galaxy, Wolf Laboratories, Pocklington, UK) with 5% CO_2_ at 37 °C. 

### 3.5. Analysis of Cell Viability

Cell viability of rat thymocytes, after cultivation period, was evaluated by CCK-8 assay as was previously described [30]. Ten µL of reaction mixture was added in each well. After 2 h of incubation, the solubilized formazan product was quantified spectrophotometrically, by using a microplate reader Perkin-Elmer (Wallac Victor^2^V, Turku, Finland). Absorbance was measured at 450 nm. For each sample, basal intensity values were subtracted from those obtained after different treatments. Absorbances were presented as ratio for comparison with control samples.

### 3.6. Measurement of Intracellular Reactive Oxygen Species (ROS) Production

A redox-sensitive probe (H2DCF-DA) was used to determine changes in overall cellular ROS levels, as described previously [31,32]. The change in fluorescence (excitation 485 nm; emission 530 nm) was measured using a Perkin-Elmer fluorimeter (Wallac Victor^2^V). For each sample, basal fluorescence intensity values were subtracted from those obtained after different treatments and results were presented as ratio of mean fluorescence intensity (MFI).

### 3.7. Determination of Mitochondrial Membrane Potential

Changes in mitochondrial membrane potential (MMP) of thymocytes, treated with camphor and borneol were evaluated by uptake of lipophilic cation Rhodamine 123 into mitochondria, as previously described [33,34]. The fluorescence of intracellular Rhodamine 123 (excitation 485 nm; emission 530 nm) was measured by Perkin-Elmer fluorimeter, as published earlier [35]. For each sample, basal fluorescence intensity values were subtracted from those obtained after different treatments and results were presented as ratio of mean fluorescence intensity (MFI).

### 3.8. Statistical Analysis

Results are presented as mean ± SD. Significant differences between the groups were analyzed with Student’s *t*-test. Avalue of p less than 0.05 was set as the significant level. 

## 4. Conclusions

In summary, we have shown that camphor at lower and borneol at higher concentrations were not able to induce toxicity in rat thymocytes, indicating their potential usage as natural health products. On the other hand, although the precise molecular mechanism of their action is not yet known, increased ROS production for camphor and decreased MMP for camphor and borneol do not represent crucial induction factors of their toxicity. 
